# Effective Characterisation of the Complete Orang-Utan Mitochondrial DNA Control Region, in the Face of Persistent Focus in Many Taxa on Shorter Hypervariable Regions

**DOI:** 10.1371/journal.pone.0168715

**Published:** 2016-12-29

**Authors:** Graham L. Banes, Biruté M. F. Galdikas

**Affiliations:** 1 Division of Biological Anthropology, Department of Archaeology and Anthropology, University of Cambridge, Cambridge, Cambridgeshire, United Kingdom; 2 Max Planck Institute for Evolutionary Anthropology, Leipzig, Germany; 3 CAS-MPG Partner Institute for Computational Biology, Shanghai, People’s Republic of China; 4 Department of Archaeology, Simon Fraser University, British Columbia, Canada; National Cheng Kung University, TAIWAN

## Abstract

The hypervariable region I (HVRI) is persistently used to discern haplotypes, to distinguish geographic subpopulations, and to infer taxonomy in a range of organisms. Numerous studies have highlighted greater heterogeneity elsewhere in the mitochondrial DNA control region, however–particularly, in some species, in other understudied hypervariable regions. To assess the abundance and utility of such potential variations in orang-utans, we characterised 36 complete control-region haplotypes, of which 13 were of Sumatran and 23 of Bornean maternal ancestry, and compared polymorphisms within these and within shorter HVRI segments predominantly analysed in prior phylogenetic studies of Sumatran (~385 bp) and Bornean (~323 bp) orang-utans. We amplified the complete control region in a single PCR that proved successful even with highly degraded, non-invasive samples. By using species-specific primers to produce a single large amplicon (~1600 bp) comprising flanking coding regions, our method also serves to better avoid amplification of nuclear mitochondrial insertions (*numts*). We found the number, length and position of hypervariable regions is inconsistent between orang-utan species, and that prior definitions of the HVRI were haphazard. Polymorphisms occurring outside the predominantly analysed segments were phylogeographically informative in isolation, and could be used to assign haplotypes to comparable clades concordant with geographic subpopulations. The predominantly analysed segments could discern only up to 76% of all haplotypes, highlighting the forensic utility of complete control-region sequences. In the face of declining sequencing costs and our proven application to poor-quality DNA extracts, we see no reason to ever amplify only specific ‘hypervariable regions’ in any taxa, particularly as their lengths and positions are inconsistent and cannot be reliably defined–yet this strategy predominates widely. Given their greater utility and consistency, we instead advocate analysis of complete control-region sequences in future studies, where any shorter segment might otherwise have proven the region of choice.

## Introduction

Predominantly maternal inheritance, large copy number, and high replication and mutation rates render mitochondrial DNA (mtDNA) especially advantageous in studies of population genetics [1]. If a female does not inter-breed or has no female offspring, and if her mtDNA haplotype does not occur elsewhere in the breeding population, her mitochondrial lineage will usually disappear [2]. Mitochondrial loci have a smaller effective population size as compared to autosomal markers, therefore, and–as mtDNA lineages are often specific to geographic areas–such loci are often used to define taxonomic units and to identify genetically distinct subpopulations [3]. Replication independent of the cell cycle [4] ensures large numbers of copies, typically up to several thousand per cell [5], facilitating mtDNA analyses from ancient or non-invasive samples when nuclear DNA quantities are insufficient or limited. High mutation rates–linked to increased susceptibility to DNA damage, higher copy-rate margin for error (reviewed in [6]) and greater oxidative stress [7]–prove especially useful in studies of small populations or those comprising few generations [8].

Actual mitochondrial DNA mutation rates are likely to vary between cell types, and the abundance and distribution of individual mutations is known to vary between species [9]. Nonetheless, it is commonly accepted that the highest mutation rates occur in the non-coding control region (or displacement ‘d’ loop, given its function in replication), which–in humans–comprises 1,122 bp of the 16,569 bp genome (*i*.*e*. 6.77%) [10]. Variable blocks may evolve four to five times faster in the control region than elsewhere in the mtDNA molecule [11,12], probably due to preferential mutational hotspots [13]. Much of this variation is concentrated within a short segment in the left domain of the control region, now commonly termed the Hypervariable Region I (HVRI) [11,14]. Given its increased and concentrated abundance of mutations, the HVRI is arguably the most commonly used mitochondrial marker in phylogenetic studies, even in the face of DNA barcoding, which typically relies on coding markers (*e*.*g*. cytochrome oxidase I) that are largely conserved within species [15]. Accordingly, the HVRI has been chosen in countless taxa to infer species, subspecies and geographic subpopulations (*e*.*g*. [16]); to determine the origins of trafficked wildlife (*e*.*g*. [17–19]); to trace the provenance of confiscated wildlife parts (*e*.*g*. [20–22]) and to identify endangered species unlawfully sold for consumption (*e*.*g*. [23]).

Despite the inherent popularity of the HVRI as a phylogenetic marker, numerous studies have drawn attention to variations elsewhere within the control region. In particular, second (*i*.*e*. HVRII) or even third (*i*.*e*. HVRIII) hypervariable regions have since been discerned in a broad range of taxa. The sizes and positions of all such hypervariable regions are often arbitrarily defined, however–even within species–with little consistency across the literature [24]. In humans, two hypervariable regions are commonly described, spanning either positions 16024–16365 (HVRI, 342 bp) and 73–340 (HVRII, 268 bp) (*e*.*g*. [25,26]) or positions 16024–16383 (HVRI, 360 bp) and 57–372 (HVRII, 316 bp) *(e*.*g*. [13,27]) in the revised Cambridge Reference Sequence [10]. Some studies consider both regions to be much longer, occasionally spanning up to 610 bp each (*e*.*g*. [28]), while others consider both to be shorter and append a compensating third hypervariable region (*i*.*e*. HVRIII) (*e*.*g* [29]). Lack of a consensus definition proves especially problematic when comparing data from multiple research efforts. Different studies that target the same regions, in the same species, will often use different primer sets to produce sequences of varying length that may not fully overlap [24].

Nonetheless, it is accepted that the HVRI is relatively short–comprising, as commonly defined in humans, up to only 32% of the complete control region. As a consequence, variation outside of the HVRI is usually overlooked, in spite of the value of such variations having been established in numerous studies. When defining only two hypervariable regions, Meyer *et al*. (1999) observed greater overall numbers of polymorphic sites in the human HVRII than in the HVRI [27]. Imes *et al*. (2012) reported a similar pattern in domestic dogs *(Canis lupis familiaris)* [30], while greater overall nucleotide diversity has been reported in the HVRII than in the HVRI of Mauritian long-tailed macaques (*Macaca fascicularis*, [31]). In some cases, variations outside of the HVRI have proved of equal or greater value in phylogeographic studies: for example, Wilkinson-Herbots *et al*. (2007) remarked that a single polymorphism in the HVRII could be used to help distinguish five major mtDNA clusters among European Caucasians [32]. Indeed, both HVRI and HVRII by any common definition exclude frequent nucleotide variants characteristic of many human populations, including transitions at 7 positions that can be used to help diagnose at least 12 established haplogroups [33]. At the very least, polymorphisms outside of the HVRI have been shown to have forensic utility in discerning distinct matrilines that might otherwise appear identical (*e*.*g*.[31,34]). It is therefore pragmatic to postulate that the complete control region–encompassing however many hypervariable regions–should be analysed as a single unit, rather than inconsistently sequencing arbitrary segments.

Orang-utans *(Pongo* spp.) are among the many species that owe their present-day taxonomy, at least partially, to studies of mitochondrial DNA. In conjunction with prior knowledge of a pericentric chromosomal inversion [35], complete mtDNA genome sequences of orang-utans from the islands of Borneo and Sumatra [36,37] were instrumental in conjunction with other mtDNA markers (*e*.*g*. [38,39]) in the decision to divide the genus into two distinct Bornean (*Pongo pygmaeus)* and Sumatran (*Pongo abelii)* species–though not without considerable debate (*e*.*g*. [40]). On the basis of short, ~245 bp sequences of a primary stretch of variation within the left domain of the mtDNA control region (*i*.*e*. a purported HVRI), Warren *et al*. (2001) later identified four geographic subpopulations on Borneo, implying that these might constitute subspecies of the Bornean orang-utan [41]. Groves (2001), on the other hand, concluded on the basis of skull morphology that only three subspecies could be defined on Borneo–and, contrary to the available genetic data–his taxonomy was apparently favoured and continues to persist [42–44]. Nonetheless, the segment amplified by Warren *et al*. (2001) continues to be the predominant mitochondrial marker used in orang-utan phylogenetic studies, though most have produced sequences of varying lengths that may not encompass the full stretch of hypervariability [45–49] (Table 1). While many studies report amplifying the ‘hypervariable region I’, the length and position of such a stretch is unknown in either species, and thus is presumably considered similar to those reported in closely related taxa. In practice, analyses of nucleotide diversity over a large number of aligned complete control-region sequences is necessary to identify and define hypervariable regions (*e*.*g*.[11,14]).

**Table 1:** Segments of the left domain of the orang-utan mitochondrial DNA control region, of varying lengths and positions, as published and analysed in prior studies. Many were cut by the original authors from longer sequences (*i*.*e*. larger PCR amplicons, containing flanking regions) prior to analysis and/or publication; only those bases derived from the control region are specified here. Nucleotide positions correspond to those of the complete Sumatran orang-utan mitochondrial DNA genome (GenBank accession code X97707; [50]) and are estimated for unpublished sequences based on the lengths noted in each manuscript. The control region spans positions 15484–16499 of the reference genome.

Publication	Accession codes	Length	Positions
Warren *et al*. (2001) [41]*	AJ391095—AJ391103; AJ391105—AJ391141	~ 245 bp	15605–15844
Jalil *et al*. (2008) [46]	EU547189—EU547201	≤ 323 bp	15516–15836
Arora *et al*. (2010) [45]	FR717918—FR717940	≤ 323 bp	15516–15836
Morrogh-Bernard *et al*. (2010) [47]	Unpublished	≤ 410 bp	15484–15893
Nater *et al*. (2013) [48]	JQ962945—JQ962972	≤ 385 bp	15484–15866
Rianti *et al*. (2015) [49]	Unpublished	≤ 422 bp	15484–15905

* Refers to original versions of sequences, prior to later updates by [45].

At the time of submitting this manuscript, only five complete control-region sequences could be found in published databanks: two from Sumatran [37] and three from Bornean [36,37]) orang-utans. The two Sumatran sequences are identical at control-region resolution. The remainder of all published control-region sequences were all considerably shorter in length, and all comprise only the left domain or parts thereof. Two segments within this domain are predominant: among Sumatran orang-utans, a ~385 bp segment [48]; among Bornean orang-utans, a ~323 bp segment [45,46] encompassed within the former.

Here, we characterise the complete mitochondrial DNA control region in Sumatran and Bornean orang-utans. We first determine the length and position of the purported hypervariable region I within the complete control region, and assess the extent to which this section was sequenced and analysed in six major published orang-utan phylogenetic studies [41,45–49] (Table 1). Rather than arbitrarily defining further hypervariable regions, we then compare the extent and utility of polymorphisms present in the remainder of the control region–all of which have been ignored to date in all prior studies of intra-island phylogeography–to those occurring in the aforementioned predominantly analysed segments (*i*.*e*. 385 bp, Sumatran; 323 bp, Bornean). We determine the minimum extent to which these variations might prove informative and useful in this and future phylogenetic studies.

## Materials and Methods

### Sample collection

Faecal samples were collected from 15 free-ranging Bornean orang-utans in Tanjung Puting National Park, Central Kalimantan, Republic of Indonesia, and genomic DNA extracted, as previously described [51]. Samples were frozen at -20°C for up to five years prior to DNA extraction. All required permits and approvals were obtained from the Indonesian State Ministry of Research and Technology (RISTEK), the Indonesian State Ministry of Forestry (PHKA), the Indonesian Institute of Sciences (LIPI), the Indonesian Agency for Natural Resource Conservation (BKSDA) and the Tanjung Puting National Park Office (BTNTP). As per the requirements of Indonesia’s domestic legislation, samples were exported to the European Union, via the United Kingdom, under permits 14459/IV/SATS-LN/2008 and 00459/IV/SATS-LN/2011 from the Convention on International Trade in Endangered Species of Wild Fauna and Flora (CITES).

Blood samples (including whole blood, serum and plasma) and tissue biopsies were collected from 18 Sumatran, 22 Bornean and 11 hybrid orang-utans, of which 6 and 5 were descended maternally from each species respectively, from zoos in the United States of America. These samples had either been banked routinely by each institution over time, or–in the case of blood products–were drawn opportunistically for use in this and other studies, either during routine medical procedures or voluntary blood-draw training. Permission was granted by each Institutional Animal Care and Use Committee or equivalent body, and approved by recommendation of the Orangutan Species Survival Plan (SSP) Steering Committee. Samples were exported from the USA under CITES permit 11US49805A/9 and imported to the European Union, via the United Kingdom, under CITES permit 477248/01-27. Genomic DNA was extracted from samples of all blood products using the standard protocol of the QIAgen DNA Blood Mini Kit, with one modification: DNA from whole blood was eluted into 400 μl, and DNA from serum and plasma into 50 μl, of Buffer AE.

Of all 66 orang-utans, at least 46 were presumed to derive from different matrilines, on the basis of institutional or reintroduction records. The remaining 20 were known to be full siblings, maternal half-siblings or matrilineal relatives of other sampled individuals, and thus were assumed to share the same mitochondrial haplotype.

### DNA amplification and sequencing

**Fig 1:**
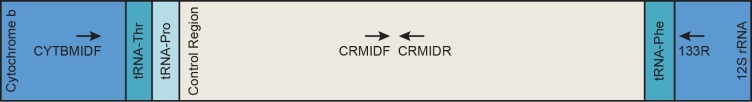
Relative position of primers used in DNA amplification and sequencing of the complete orang-utan mitochondrial DNA control region. The sizes of each region in this schematic are proportional to their sizes in the orang-utan mitochondrial genome.

Improving on prior studies, we amplified the complete orang-utan mitochondrial DNA control region in a single polymerase chain reaction (PCR), in a protocol that proved successful even for highly degraded and low-quantity faecal- and serum-derived DNA extracts. We designed the orang-utan-specific primers CYTBMIDF (5-CAA TCC TAC GAT CCG TCC CC-3) and 133R (5-CGG GGA TGC TTG CAT GTG TAA C-3) to anneal to the cytochrome b and 12S rRNA regions respectively, on opposite sides of the control region (Fig 1). By targeting the complete control region in a single reaction, and by using species-specific primers, we intended to mitigate the risk of amplifying nuclear mitochondrial insertions (‘*numts’*), which are known to be paralogous to orang-utan mitochondrial DNA [52] and which have proven problematic in phylogenetic studies of a range of other hominoid taxa [52]. We chose to use the Expand Long-Range dNTPack (Roche) to amplify the target region, which typically proved successful with only 1 μl template DNA irrespective of the source material. Though we successfully amplified the region using lower-fidelity enzymes and master mixes, such reagents required considerable optimization between reactions and, in the case of faecal samples, unsustainably large quantities (up to 10 μl) of template DNA in short supply from wild individuals.

Each 25 μl reaction comprised 5 μl 5x Expand Long Range Buffer with MgCl_2_ (12.5 mM), 1.25 μl dNTPs (10 mM each), 1.5 μl 100% DMSO, 0.75 μl each primer (10 μM), 0.35 μl Expand Long Range Enzyme Mix (5 U/μl), 14.4 μl ddH_2_O and 1 μl DNA extract. Thermal cycling conditions were as follows: initial denaturation at 92°C for 2 minutes; 10 cycles of 92°C for 10 seconds, 60°C for 15 seconds and 68°C for 2 minutes 30 seconds; 15–20 cycles of 92°C for 10 seconds, 60°C for 15 seconds and 68°C for 2 minutes 30 seconds (plus 20 seconds per cycle); and a final extension at 68°C for 7 minutes, followed by a 4°C hold. To reduce the risk of PCR error, we amplified DNA from each orang-utan twice, in two independent PCR reactions, each with template DNA derived from different DNA extracts.

PCR products were electrophoresed on 1% TAE agarose gels, and only those yielding single bright bands of the expected size were subject to DNA sequencing, using an ABI 3730*xl* DNA Analyzer with integrated KB basecaller under BigDye Terminator v3 cycling conditions (Applied Biosystems). Due to the limitations of the Sanger method in producing sufficiently long sequences, we ‘primer walked’ each pair of reactions with two different sets of primers, producing overlapping sequence contigs for each DNA extract. The first product of each pair was sequenced twice in the forward direction, using the primers CYTBMIDF and CRMIDF (5-CCC CTC AGT TAG TGG TCC CT-3, this study). The second product was sequenced twice in the reverse direction, using the primers CRMIDR (5-GGA GCG AGG AGA GTA GCA CT-3, this study) and 133R (Fig 1). Each base was therefore confirmed at least twice independently, from overlapping sequences generated from two separate reactions each from different DNA extracts.

### Sequence assembly

Sequence contigs were assembled by mapping reads to a reference sequence of the complete Sumatran orang-utan mitochondrial genome, in GENEIOUS 8.1.8 [53] (GenBank accession code X97707; [37]). Chromatograms were manually checked in all cases where a base was called with lower than 99% probability of being correct. We used RDP4 [54] to check for recombination among sequences, *sensu* [55], as a means of identifying *numts*. Consensus sequences were collapsed into haplotypes and aligned using MUSCLE [56], with 3 of the 4 unique complete control-region sequences available in GenBank at the time of analysis: accession codes X97707 (*P*. *abelii*), X97709 and X98472 (both *P*. *pygmaeus*) (all [37]). As the published sequence of Horai *et al*. (1992) [36] (accession code D38115) features an 81 bp deletion not observed in these other published sequences, and given that another of those authors’ sequences was found to be a composite of several from multiple DNA sources [57], we chose to exclude this sequence.

### Sequence analysis

Hypervariable regions were visualised by plotting average nucleotide diversity among the alignment of all sequences, computed in DnaSP 5.10.01 [58] via sliding window and considering all alignment gaps across all sites. We determined the nucleotide positions of each region by first discerning those of conserved regions, as identified by inferring a conservation threshold given the observed number of segregating sites *(S)*.

To demonstrate the forensic utility of progressively longer sequences, we cut our alignment to the various lengths of those published and analysed in six prior studies: 244 positions [41]; 325 positions [45,46]; 387 positions [48]; 409 positions [47] and 422 positions [49] (for nucleotide positions, see Table 1). In the case of the latter two studies, for which the authors chose not to release their sequences, we cut the alignment based on the length of the analysed sequences specified in each manuscript. We computed the number of haplotypes (*h*), number of polymorphic sites and total number of mutations, and mean number of nucleotide/pairwise differences (*k*) among alignments of each length in DnaSP 5.10.01 [58].

To assess the phylogeographic utility of those nucleotide positions not typically sequenced in Bornean orang-utans, we created two alignments comprising all of our novel complete control-region sequences, plus four chimpanzee *(Pan troglodytes)* sequences from GenBank to serve as an outgroup. For each, we either: 1) re-aligned the sequences with those derived from Bornean orang-utans of known geographic origin [45], and cut the alignment to the length of the predominantly analysed 323 bp segment; or 2) excised the 323 bp segment predominantly analysed for Bornean orang-utans, concatenated the remaining bases, and re-aligned the nucleotides. We repeated this procedure for Sumatran orang-utans, with published sequences from individuals of known origin on Sumatra [48], instead cutting the alignment to the 385 bp segment predominantly analysed in this species. We inferred phylogenetic trees from all alignments, and from an alignment of only all complete control-region sequences, with which to compare the consistency of inferred evolutionary relationships. Accession codes for published sequences used in these analyses are indicated in the S1 Table.

We used jModelTest 2.1.7 [59], computing likelihood calculations using Phyml [60] to determine the best model of nucleotide substitutions for alignments under corrected Akaike Information Criteria (AICc). Trees were estimated using MrBAYES 3.2.6 [61] implementing Metropolis-coupled Markov chain Monte Carlo (MCMCMC) to approximate posterior probabilities, with four chains swapped at a frequency of 0.2. Analyses were run for a maximum of 10,000,000 generations from a randomly generated tree, sampling every 100 generations. The first 25% of trees generated before convergence occurred were discarded. Resulting trees were drawn as cladograms; nodes were labelled with posterior probabilities.

## Results

Complete control-region sequences were successfully generated from all 66 samples. In some cases, it was necessary to perform further DNA extractions–particularly from faecal samples–in order to yield sufficient genomic DNA for amplification. In all such cases, however, we were unable to amplify even short segments of the purported HVRI [46] using the initial DNA extracts: thus, the DNA was either of sufficient quality for complete control-region amplification, or entirely insufficient for amplification at all. In no circumstances could we amplify only shorter segments of the purported HVRI, but not the complete control region, in a single PCR.

The resulting 36 complete control-region haplotypes ranged from 999–1016 bp in length (Sumatran: 1011–1016 bp; N = 13; Bornean: 999–1000 bp, N = 23), and have since been submitted to GenBank (accession codes KX427542—KX427574). Three update shorter sequences previously generated from the same individuals in our prior study [18], and retain their original accession codes (KU523975-KU523977). One sequence (haplotype S4) proved identical at this resolution to a published full-genome sequence in GenBank (accession code X97708; [37]), known to have derived from a different matriline. Heteroplasmy was not observed in any sequence, nor were any bases heterozygous in sequence contigs. We did not detect recombination in any sequence, and those from maternally related individuals proved identical as expected, irrespective of sample type. This considered–and given our diligence in exercising all feasible precautions–we have no reason to believe our sequences feature nuclear mitochondrial insertions.

### Presence and position of hypervariable regions

**Fig 2:**
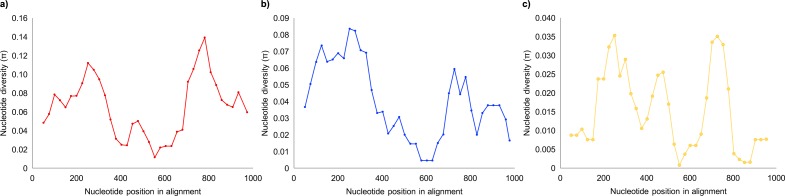
Nucleotide diversity throughout the complete orang-utan mitochondrial DNA control region. Diversity was computed for alignments comprising, a) all 38 sequences (1022 sites), b) 13 sequences of Sumatran maternal ancestry (1018 sites), and c) 25 sequences of Bornean maternal ancestry (1002 sites). Data were computed under the sliding window method, using a window length of 100 sites and a step size of 25 sites, excluding sites with gaps.

An alignment of all 35 novel sequences with those three previously published [37] comprised 1022 nucleotide positions. On the basis of sliding window analysis, we identified a primary stretch of variation, *i*.*e*. HVRI, spanning at least the first 376 sites (≤374 bp) and consistent among sequences of Bornean and Sumatran origin (Fig 2). When using the complete Sumatran orang-utan mtDNA genome of Xu and Arnason (1996) as a reference for sequences from both species (accession code X97707), we consider the HVRI to span at least positions 15484–15855. When referenced against the two pre-existing complete control-region sequences, the HVRI spans at least positions 1–372 in Sumatran orang-utans (accession code X97708) and at least positions 1–374 in Bornean orang-utans (accession code X97709). As defined here, the orang-utan HVRI aligns well with the human revised Cambridge Reference Sequence, from positions 16024–16400 (accession code NC_012920) [10].

Of all prior studies considered, only three amplified the complete hypervariable region I as defined herein [47–49]. However, the authors of two of these studies [47,49] elected not to submit their sequences for publication (*e*.*g*. in GenBank), though the latter manuscript comprised a detailed description of SNP positions. As a consequence, the only sequences presently available in GenBank that span the entire HVRI are those of Xu and Arnason (1996) (complete control region, N = 3), that of Horai *et al*. (1992) and of disputed quality (complete control region, N = 1) [36], and those of Nater *et al*. (2013) (≤ 385 bp, N = 28). All other published sequences span incomplete segments.

Though we identified further hypervariable stretches, their positions were inconsistent between Bornean and Sumatran sequences. Among those of Bornean origin, three especially hypervariable stretches were apparent, versus only two among those of Sumatran ancestry (Fig 2). Such inconsistencies could be the product of sample size, or sampling unrepresentative of the entire geographic range of each species. Having questioned the value of describing arbitrary hypervariable regions, and to avoid contributing further confusion to the literature, we chose not to formally define second or third hypervariable regions. Instead, we consider the value and utility of complete control-region sequences.

### Nucleotide diversity in progressively longer control-region segments

The number of haplotypes, the number of polymorphic sites, the total number of mutations and the average number of nucleotide differences correlated positively with sequence length (Table 2). Only complete control-region sequences could discern the full total of 38 haplotypes and complete range of nucleotide diversity. Complete control-region sequences contained more than twice the number of polymorphic sites, total mutations and nucleotide differences *(k)* than any of the shorter segments previously studied.

**Table 2:** Diversity indices computed from three alignments of 35 novel and unique complete control-region sequences generated in this study, plus three published complete control-region sequences, for all sequences and for those of Bornean or Sumatran orang-utans only. Alignments were trimmed to the lengths of various shorter segments sequenced in prior studies.

	No. of sites	Extent of CR	Positions	No. of haplotypes, *h*	No. of polymorphic sites (total no. of mutations)		Average no. nucleotide differences, *k*
**All orang-utans (38 sequences):**							
Warren *et al*. (2001) [41]	244	24%	104–381	22	64	(73)	20.394
Jalil *et al*. (2008); Arora *et al*. (2010) [45,46]	325	32%	033–355	28	81	(90)	25.515
Nater *et al*. (2013) [48]	387	38%	001–386	29	88	(97)	27.733
Morrogh-Bernard *et al*. (2010) [47]	409	40%	001–409	29	88	(97)	27.686
Rianti *et al*. (2015) [49]	422	41%	001–422	29	89	(98)	27.879
This study–complete CR	1022	100%	001–1000	38	186	(200)	62.950
**Sumatran orang-utans (13 sequences):**							
Warren *et al*. (2001) [41]	242	24%	104–381	8	37	(37)	15.744
Jalil *et al*. (2008); Arora *et al*. (2010) [45,46]	323	32%	033–355	9	47	(47)	20.333
Nater *et al*. (2013) [48]	385	38%	001–386	9	47	(47)	20.333
Morrogh-Bernard *et al*. (2010) [47]	407	40%	001–409	9	53	(53)	23.026
Rianti *et al*. (2015) [49]	420	41%	001–422	9	54	(54)	23.487
This study–complete CR	1018	100%	001–999	13	90	(91)	39.744
This study–oft-ignored bases	633	62%	387–999	9	37	(38)	16.641
**Bornean orang-utans (25 sequences):**							
Warren *et al*. (2001) [41]	244	24%	104–381	14	25	(27)	5.430
Jalil *et al*. (2008); Arora *et al*. (2010) [45,46]	323	32%	033–355	19	31	(33)	6.310
Nater *et al*. (2013) [48]	386	39%	001–386	20	34	(36)	6.770
Morrogh-Bernard *et al*. (2010) [47]	408	41%	001–409	20	34	(36)	6.770
Rianti *et al*. (2015) [49]	421	42%	001–422	20	34	(36)	6.770
This study–complete CR	1002	100%	001–999	25	67	(70)	14.427
This study–oft-ignored bases	678	68%	001–032; 356–999	21	36	(37)	8.117

### Phylogeographic utility of predominantly analysed segments, versus the remainder of the control region

**Fig 3:**
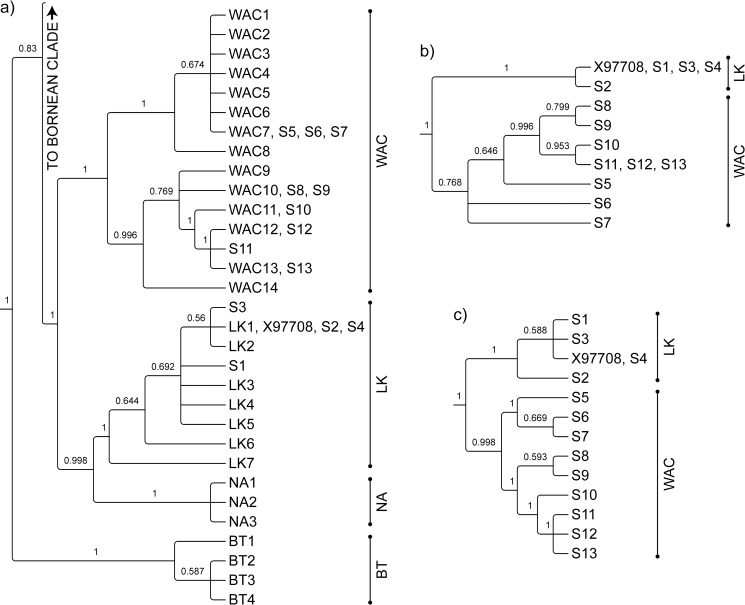
**Evolutionary relationships for alignments of DNA sequences spanning a) 385 bp of the left domain of the Sumatran orang-utan mitochondrial DNA control region, as predominantly analysed in prior phylogenetic studies; b) all remaining nucleotides in the control region; c) all nucleotides of the control region.** Sequences from orang-utans of known geographic origin are prefixed WAC (West Alas Cluster), LK (Langkat), NA (North Aceh) or BT (Batang Toru); novel sequences are prefixed 'S'; other published sequences are named with their accession numbers. Sequences from Bornean orang-utans and chimpanzees, used as outgroups, are not shown. Published sequences used in this inference are detailed in S1 Table.

**Fig 4:**
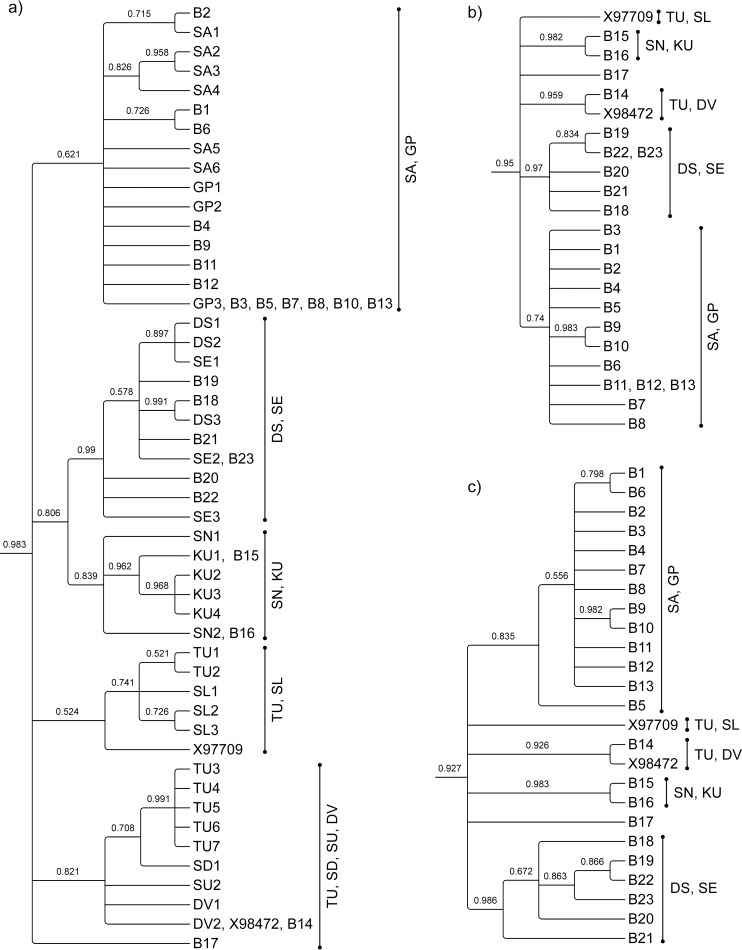
**Evolutionary relationships for alignments of DNA sequences spanning a) 323 bp of the left domain of the Bornean orang-utan mitochondrial DNA control region, as predominantly analysed in prior phylogenetic studies; b) all remaining nucleotides in the control region; c) all nucleotides of the control region.** Sequences from orang-utans of known geographic origin are prefixed SA (Sabangau), GP (Gunung Palung), DS (Danau Sentarum), SE (Semenggoh), SN (Sangatta), KU (Kutai), TU (Tuanan), SL (Sungai Lading), SD (Sandakan) and DV (Danum Valley); novel sequences are prefixed 'S'; other published sequences are named with their accession numbers. Sequences from Sumatran orang-utans and chimpanzees, used an outgroup, are not shown. Published sequences used in this inference are detailed in S1 Table.

By inferring evolutionary relationships from the predominantly analysed segment (*i*.*e*. 385 bp), we inferred our sequences of Sumatran maternal origin derived ancestrally from two of the four subpopulations previously discerned on Sumatra [48] (Fig 3). These sequences were correctly resolved into distinct clades on a tree inferred only from all remaining control-region nucleotides. Thus, the remainder of the control region is phylogeographically informative, in that polymorphisms can distinguish at least two subpopulations. Similarly, sequences of Bornean origin were concordantly resolved into the same five different clades, representing different geographic subpopulations, using either the predominantly analysed segment (*i*.*e*. 323 bp) or all remaining control-region nucleotides (Fig 4). Polymorphic sites in the remainder of the Bornean orang-utan control region are therefore at least as informative as those in the predominantly analysed segment.

## Discussion

It is evident that there is considerable variation in orang-utans, in one or more additional hypervariable regions, in those areas of the control region that are typically ignored. Despite this, the hypervariable region I is broadly used as a representative sample of the control region in numerous taxa, on the understanding that it comprises the most abundant and useful of all polymorphisms for most phylogenetic applications. On this basis, prior orang-utan phylogenetic studies have demonstrably underestimated true control-region diversity, in choosing to focus only on the hypervariable region I or shorter segments thereof. It is probable that fewer haplotypes were characterised in such studies, as matrilineally unrelated individuals–that could have been distinguished by complete control-region sequences–may have been clustered together. Furthermore, as polymorphic sites in the remainder of the control region at least mirror the phylogeographic signals of the shorter and predominantly sequenced segments, it is possible that—with broader sampling—such sites could prove diagnostic of further geographic substructuring.

These observations are not intended as criticism of any previous study: several in particular have comprised especially monumental efforts to collect and analyse samples from across the entire geographic range of both species (i.e. [41,45,48]). Their analyses of short segments of the HVRI have significantly improved our understanding of orang-utan phylogeography, and all incorporated other markers–including at autosomal microsatellite and sex-chromosomal loci–from which to draw their conclusions. Indeed, in the case of orang-utans, it is unlikely that polymorphisms elsewhere in the control region would prove to be of taxonomic importance: studies at much higher resolutions, incorporating full mtDNA genome and nuclear-genome-wide variations, are already underway or published by other research groups, and are needed to explain the abundant idiosyncrasies observed in orang-utan taxonomy. Bornean orang-utan subspecies are known not to be reciprocally monophyletic based on mtDNA [41,45], though this may–to some extent–be the result of male dispersal or the unintentional translocation and reintroduction of non-native individuals [18,45,46]. More notably, through phylogenetic analysis of mtDNA, one Sumatran subpopulation has been shown to be more closely related to orang-utans on Borneo than to others on Sumatra [48]. Nonetheless, complete orang-utan control-region sequences still have superior value: not least in their ability to distinguish distinct individuals, from different matrilines, that might otherwise share haplotypes at lower resolutions. Insertion/deletion polymorphisms are comparatively sparse in the remainder of the control region, as compared to the HVRI: in such instances, generating the complete control region provides substantially more data for phylogenetic analysis and reduces the potential for severe alignment errors. A consistent 15 bp deletion towards the end of the Bornean orang-utan control region could also prove useful, in facilitating their rapid discrimination from Sumatran sequences simply through visualization of product size via gel electrophoresis. Current methods utilize restriction enzymes applied to PCR products, and are therefore less feasible in field laboratories or in those in developing countries [62].

Our findings are relevant to all future control-region studies, however, irrespective of taxa. As mitochondrial genome structure is largely conserved, mutational hotspots can be expected throughout the complete control region in general [1,13]. Focusing only on short, arbitrarily defined and purported hypervariable segments has therefore potentially underestimated genetic diversity and population substructuring in a range of prior studies. This might occur when such segments are incorrectly considered to be broadly representative of complete control- region variation: when in fact, as in the case of orang-utans, useful polymorphisms are located elsewhere in the region that could be used to help distinguish distinct subpopulations or matrilines. The extent to which this might have occurred is unknown, as–in the clear majority of species–only purported hypervariable regions have been sequenced, rather than the complete control region: the abundance, value and utility of polymorphisms occurring elsewhere in such cases is therefore yet to be characterised. The issue might also predominate when aligning sequences of the ‘same’ region, produced by different studies, when each defines the length and position of this region differently. For successful phylogenetic analysis, such alignments must be cut to the length of the shortest sequence, encompassing only the narrowest definition. Consequently, genetic diversity within the author’s definition of the HVRI could be underestimated, having partially discarded the nucleotides. This will continue to be problematic, even when using complete control-region sequences: though we generated such sequences from orang-utans in our prior study, it was necessary to cut these to the shorter lengths of published sequences to infer their subspecies affiliations [18]. In that scenario–discounting the benefits of avoiding amplification of numts, or extraneous human DNA–there was no superior value to complete control-region sequences, as most additional nucleotides were simply discarded.

The need for consistency in future studies is therefore paramount, yet segments such as the ‘hypervariable region’ cannot be consistently defined. Their lengths and positions must be determined through analysis of numerous sequences, using the sliding window approach adopted herein, or more complex change-point detection methods. As sequences are added and removed from the sample, particularly from genetically distinct individuals or populations, the lengths and positions of hypervariable and conserved regions will change. By contrast, the position of the control region is well-defined and conserved across taxa, and its length–excluding insertions and deletions–is typically uniform within species. Complete control-region sequences from multiple studies can therefore be aligned and analysed with no such ambiguity. Given the affordability and ease at which one can now amplify complete control-region sequences, even from highly degraded DNA, we therefore strongly advocate their use in all future studies where a shorter segment might otherwise have proven the locus of choice.

We acknowledge that the control region itself is simply a small segment of a larger, variable genome. In their analysis of 241 complete human mitochondrial genomes, for example, Coble *et al*. (2004) found that 18 haplotypes at the resolution of the HVRI and HVRII increased to 105 over the entire genome–though, having chosen only samples from European Caucasians, it is unclear to what extent these variations might be phylogeographically informative [63]. Nonetheless, complete mitochondrial genome sequencing continues to be unaffordable for the vast majority of laboratories: such technology is as far in the distant future for cost-stretched Western research groups as it is for those in developing countries; particularly for researchers who study less charismatic fauna for which endeavours are comparatively poorly funded. For those without next-generation sequencing (NGS) capability, primer-walking and Sanger sequencing of mitochondrial genomes is possible, but still expensive–particularly when studies necessitate a large number of individual samples. Though the price of NGS continues to decline, the costs of library preparation are still insurmountable for many research groups, and thus, in both cases, only shorter segments such as the hypervariable region I are feasible for analysis.

We do not underestimate the value of nuclear markers, and we appreciate that–given limited funds–some might opt to sequence such a locus in addition to a short control-region segment, rather than devoting their additional budget to encompassing the complete control region. We do not consider these options to be equal, however. The costs of adding an independent marker are considerably greater, given that one requires more reagents and primers to perform additional PCRs. In contrast, it is not particularly more expensive to amplify and sequence the complete control region in a single PCR than to do so only for any shorter segment: the only supplementary cost is a second pair of sequencing reads. Given this minimal increase, and the fact that the procedure is no more complex or labour intensive, we suggest that complete control-region sequencing becomes the new *de facto* standard–and the new ‘bare minimum’–in favour of targeting any shorter segment thereof. Indeed, based on our findings, we find it hard to reconcile that any researcher would intentionally generate anything shorter.

Our view is shared by the forensic science community, for whom analysis of complete control-region sequences has long been the norm: guidelines to do so were introduced in 2007 [33], and–since 2013 –a leading journal, *Forensic Science International*: *Genetics*, has declined to accept submissions reliant only on partial control-region sequences [64]. The zoological community is yet to catch up, however. Having compared and demonstrated their superior utility and potential in a non-human animal species, we hope to encourage the use of at least complete control-region sequences in future studies, where a short and arbitrary segment thereof might otherwise have proven the exclusive locus of choice.

## Supporting Information

S1 Table Accession codes of published sequences used in phylogenetic analyses. (DOCX)
