# Supplementary material for: Effective Characterisation of the Complete Orang-Utan Mitochondrial DNA Control Region, in the Face of Persistent Focus in Many Taxa on Shorter Hypervariable Regions
Source: PLoS One. 2016 Dec 29;11(12):e0168715. doi: 10.1371/journal.pone.0168715 (PMC5199090; doi:10.1371/journal.pone.0168715)
Supplement: S1 Table — (DOCX) [file pone.0168715.s001.docx]

**S1 Table** Accession codes of published sequences used in phylogenetic analyses.

| **Identifier** | **Accession code** | **Species** | **Reference** |
| --- | --- | --- | --- |
| - | HM068576 | *Pan troglodytes* | Bjork *et al.* (2011) |
| - | HM068579 |  |  |
| - | GU112742 |  | Stone *et al.* (2009) |
| - | JF727204 |  | Fischer *et al.* (2011) |
| - | JF727205 |  |  |
| X97709 | X97709 | *Pongo pygmaeus* | Xu and Arnason (1996) |
| X98472 | X98472 |  |  |
| X97708 | X97708 | *Pongo abelii* |  |
| WAC1 | JQ962946 | *Pongo abelii* | Nater *et al.* (2013) |
| WAC2 | JQ962953 |  |  |
| WAC3 | JQ962947 |  |  |
| WAC4 | JQ962952 |  |  |
| WAC5 | JQ962965 |  |  |
| WAC6 | JQ962972 |  |  |
| WAC7 | JQ962945 |  |  |
| WAC8 | JQ962964 |  |  |
| WAC9 | JQ962968 |  |  |
| WAC10 | JQ962967 |  |  |
| WAC11 | JQ962970 |  |  |
| WAC12 | JQ962966 |  |  |
| WAC13 | JQ962971 |  |  |
| WAC14 | JQ962969 |  |  |
| LK1 | JQ962955 |  |  |
| LK2 | JQ962958 |  |  |
| LK3 | JQ962957 |  |  |
| LK4 | JQ962959 |  |  |
| LK5 | JQ962960 |  |  |
| LK6 | JQ962954 |  |  |
| LK7 | JQ962956 |  |  |
| NA1 | JQ962962 |  |  |
| NA2 | JQ962961 |  |  |
| NA3 | JQ962963 |  |  |
| BT1 | JQ962949 |  |  |
| BT2 | JQ962950 |  |  |
| BT3 | JQ962951 |  |  |
| BT4 | JQ962948 |  |  |
| GP1 | AJ391105.2 | *Pongo pygmaeus* | Warren *et al.* (2001); Arora *et al.* (2010) |
| GP2 | AJ391107.2 |  |  |
| GP3 | AJ391106.2 |  |  |
| DS1 | AJ391101.2 |  |  |
| DS2 | AJ391103.2 |  |  |
| SE1 | AJ391123.2 |  |  |
| DS3 | AJ391100.2 |  |  |
| SE2 | AJ391121.2 |  |  |
| SE3 | AJ391125.2 |  |  |
| SN1 | AJ391110.2 |  |  |
| KU1 | AJ391133.2 |  |  |
| KU2 | AJ391136.2 |  |  |
| KU3 | AJ391137.2 |  |  |
| KU4 | AJ391134.2 |  |  |
| SN2 | AJ391109.2 |  |  |
| SD1 | AJ391120.2 |  |  |
| SU1 | AJ391117.2 |  |  |
| SA1 | FR717932 |  |  |
| SA2 | FR717934 |  |  |
| SA3 | FR717929 | *Pongo pygmaeus* | Warren *et al.* (2001); Arora *et al.* (2010) |
| SA4 | FR717931 |  |  |
| SA5 | FR717928 |  |  |
| SA6 | FR717933 |  |  |
| TU1 | FR717921 |  |  |
| TU2 | FR717922 |  |  |
| SL1 | FR717926 |  |  |
| SL2 | FR717925 |  |  |
| SL3 | FR717927 |  |  |
| TU3 | FR717919 |  |  |
| TU4 | FR717920 |  |  |
| TU5 | FR717923 |  |  |
| TU6 | FR717924 |  |  |
| TU7 | FR717918 |  |  |
| DV | FR717936 |  |  |
| DV2 | FR717935 |  |  |
